# Pertussis resurgence: epidemiological trends, pathogenic mechanisms, and preventive strategies

**DOI:** 10.3389/fimmu.2025.1618883

**Published:** 2025-07-10

**Authors:** Yaping Sheng, Shengjie Ma, Qi Zhou, Jiancheng Xu

**Affiliations:** ^1^ Department of Laboratory Medicine, Center of Infectious Diseases and Pathogen Biology, The First Hospital of Jilin University, Changchun, Jilin, China; ^2^ Department of Gastrocolorectal Surgery, General Surgery Center, The First Hospital of Jilin University, Changchun, Jilin, China; ^3^ Department of Neonatal, Children’s Medical Center, The First Hospital of Jilin University, Changchun, Jilin, China

**Keywords:** Bordetella pertussis, pertussis resurgence, epidemiology, pathogenic mechanisms, vaccines, waning immunity, preventive strategies

## Abstract

Pertussis, also known as whooping cough, is a highly contagious acute respiratory infection primarily caused by *Bordetella pertussis*. Although this disease can occur at any age, infants and young children remain the most vulnerable to severe illness and mortality. Moreover, epidemiological trends indicate a notable shift in the incidence of pertussis over time, with an increasing number of reported cases in adolescents and adults. During the 1950s, the widespread implementation of whole-cell pertussis (wP) vaccines significantly reduced the incidence and mortality associated with pertussis. Despite their effectiveness, the frequent adverse reactions linked to wP vaccines prompted a shift towards the utilization of acellular pertussis (aP) vaccines, which have a lower reactogenicity. However, over the past two decades, several countries with a high coverage of aP vaccines have experienced a notable rise in the incidence of pertussis, a phenomenon called pertussis resurgence. The causes of this resurgence are multifactorial and highly complex. Notably, the peak incidence of pertussis has shifted from the infant population to adolescents and adults, who now serve as the primary sources of infection in infants. Such a shift raises critical concerns regarding the current and future control of pertussis. The lack of comprehensive understanding of its pathogenesis is a significant contributing factor to this public health challenge. Although extensive research on the pathogenesis of pertussis has been conducted, it remains an issue without appropriate animal models that effectively replicate the symptomatology commonly observed in human cases. This review provides an overview of *B. pertussis* epidemiology and recent pathogenesis advances. It further analyzes the potential causes and contributing elements responsible for the resurgence of pertussis. Lastly, the review proposes evidence-based strategies aimed at enhancing public awareness and implementing effective measures to prevent the risk of unexpected outbreaks.

## 1 Introduction

Pertussis, commonly known as whooping cough, is an acute respiratory infection that is highly contagious and mainly caused by the Gram-negative bacterium *Bordetella pertussis*. Characterized by severe and persistent coughing episodes lasting up to 2–3 months, this disease can lead to complications such as pulmonary hypertension, pneumonia, and cerebral hemorrhage, particularly in infants, resulting in high mortality (1, 2) Prior to the advent of vaccines, pertussis was a common cause of morbidity and mortality in children (3). The introduction of whole-cell pertussis (wP) vaccines in the 1950s led to a significant decline the global incidence and mortality (4). However, due to frequent adverse reactions linked to wP vaccines, they were eventually supplanted by acellular pertussis (aP) vaccines, which have a lower reactogenicity (5, 6). In the past two decades, there has been an upward trend in the global coverage of infants receiving three doses of the aP vaccine. By 2023, this coverage had risen to approximately 84% (7). Despite achieving high vaccination coverage globally, the resurgence of pertussis has emerged as a global phenomenon, with documented occurrences in numerous countries worldwide (8, 9). For instance, in the United States, the annual number of reported cases increased from 4,000 in the 1980s to 48,277 in 2012, with an incidence rate exceeding 10 cases per 100,000 population, the highest level since the mid-1950s (10). Similarly, pertussis incidence has shown an upward trend in China, increasing from 0.13 per 100,000 individuals in 2013 to 2.15 per 100,000 in 2019 (11). Most epidemiological data regarding pertussis in China derived from a passive reporting system, the National Notifiable Infectious Disease Surveillance System, and under-reporting was considerable due to challenges related to diagnostic capabilities and the completeness of reporting processes. With the improvement of the reporting system, there has been an increase in the reporting of pertussis cases (11). In addition, the resurgence of pertussis in the United Kingdom has been observed, with incidence levels not documented since the 1980s (12). Research examining the epidemiology of pertussis in Asian nations indicates that the disease is circulating among adolescents and adults (13). Surveillance capacities for pertussis are insufficient in low- and middle-income countries (LMICs), but existing evidence suggests that these countries may also be experiencing a resurgence of the disease (14). The COVID-19 pandemic in 2020–2021 resulted in a significant decrease in the incidence of pertussis cases. However, a notable increase in reported cases was documented after the pandemic (15–17). Additionally, the epidemiology and transmission patterns of pertussis have undergone significant changes, with a rising incidence among adolescents and adults, who have increasingly been identified as primary reservoirs of infant infection. Moreover, infants, especially those who are too young to be vaccinated, often showing the most severe symptoms and even mortality (18). Therefore, pertussis remains a significant public health issue. Many hypotheses have been proposed to elucidate the resurgence of pertussis. This review provides a comprehensive analysis of the pathogenesis and epidemiology of pertussis as well as the causes of its resurgence and the response measures implemented. This information will enhance our understanding of pertussis so that more effective strategies can be developed to control its spread.

## 2 Epidemiology trends

### 2.1 Global epidemiology

**Figure 1:**
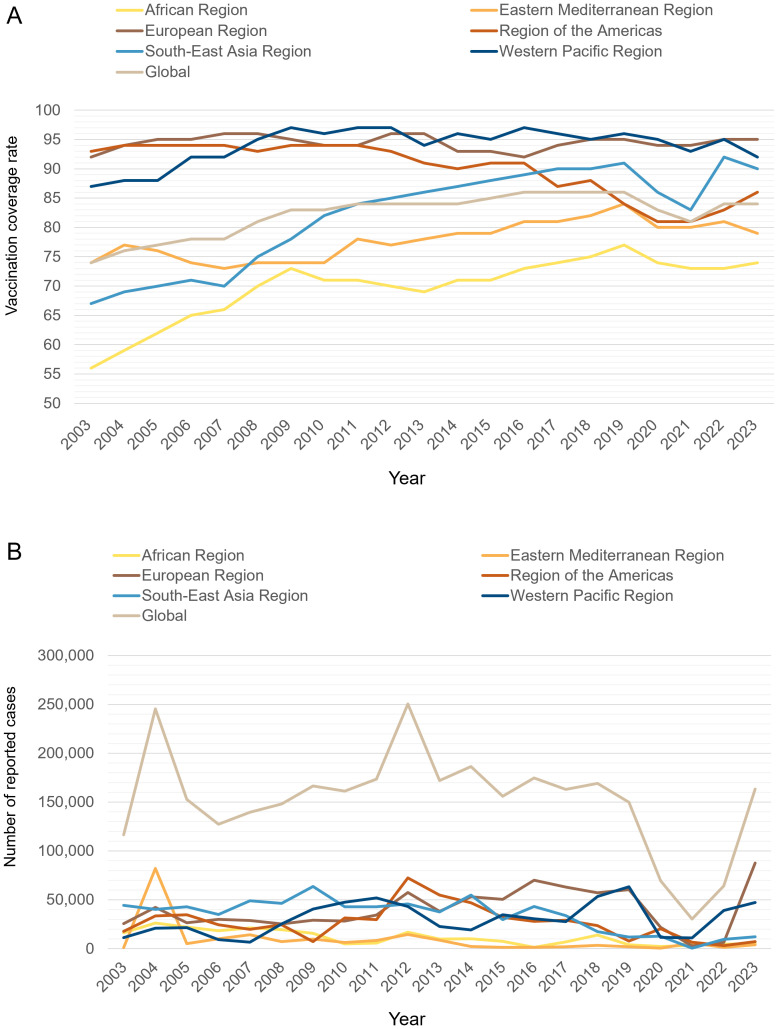
**(A)** Diphtheria tetanus toxoid and pertussis (DTP3) immunization coverage among 1-year-old children from 2003 to 2023 across the 6 WHO regions and globally (%). The x-axis represents the year, and the y-axis represents the immunization coverage rate for DTP3 among 1-year-old children (%). **(B)** Reported cases of pertussis from 2003 to 2023 across the 6 WHO regions and globally. The x-axis represents the year, and the y-axis represents the reported number of pertussis cases. Data were collected from the WHO website.

Pertussis has been documented since the Sui Dynasty in China (518–608 AD), where it was categorized as a pediatric disease and referred to as “hundred-day cough” (19). The first documented epidemic took place in Paris in 1578. Since then, localized infections and periodic outbreaks have been reported worldwide (20). By the 19th century, pertussis was widely recognized as one of the deadliest diseases affecting humans, notably contributing to high rates of infant mortality (21, 22). According to the World Health Organization (WHO), over 3 million children died from pertussis annually worldwide before 1970 (23). In the United States alone, approximately 73,000 deaths were recorded between 1922 and 1931, with the majority occurring in infants (24). The introduction of wP vaccines in the 1950s and their widespread implementation, followed by the expansion of immunization programs in the 1970s, contributed to a substantial decline in its global incidence and mortality (25). In the United States, the annual pertussis incidence declined sharply from 157 per 100,000 people in the early 1940s to fewer than 1 per 100,000 by 1973 (4). Similarly, Canada’s average incidence dropped from approximately 160 cases per 100,000 people between 1934 and 1943 to about 11 cases per 100,000 between 1974 and 1983 (26). However, due to concerns over the reactogenicity of wP vaccines and parental fears regarding potential severe neurological complications or fatal outcomes, most industrialized countries transitioned to less reactogenic aP vaccines (6, 27). However, outbreaks of pertussis have continued to emerge in the era of aP vaccines. Periodic epidemics occur every 3–5 years, similar to those observed before widespread vaccination (28). Recent data from the WHO indicate that global coverage of three doses of the diphtheria, tetanus, and pertussis vaccine (DTP3) among 1-year-old children (%) has consistently surpassed 80% over the past decade (7). However, this aggregate figure conceals significant gaps in pertussis immunization. In 2023, the European Region achieved 95% DTP3 coverage, whereas the African region managed only 74% (**Figure 1A**) (7). Furthermore, approximately 94% of children in high-income countries completed the DTP3 in 2023. However, in LMICs, factors like limited income and challenges in accessing healthcare make it difficult to achieve high vaccination coverage (7, 14). Globally, reported cases experienced significant peaks around the years 2004 and 2012, followed by a trend of moderate declines. Notably, a sharp reduction in cases was recorded during the COVID-19 pandemic. However, by 2023, global case numbers rebounded markedly. Between 2012 and 2014, both the European region and region of the Americas experienced notable peaks in case numbers. Following this period, there was a marginal decline; however, the overall incidence rates remained relatively elevated. By 2023, the European region saw a substantial increase in cases, marking the highest levels recorded in nearly ten years. In contrast, the African and Eastern Mediterranean region exhibited comparatively low case numbers across the observed period. This phenomenon may be indicative of underreporting, which could be attributed to the limitations of surveillance infrastructure in these areas (**Figure 1B**) (8, 29). Data from various countries also indicate a significant increase in pertussis cases. In fact, the baseline incidence of pertussis in the United States rose between 2000 and 2016 (30). Furthermore, the resurgence of pertussis in the United Kingdom has been observed, with incidence levels not documented since the 1980s (12). Surveillance capacities for pertussis are insufficient in LMICs, but existing evidence suggests that these countries may also be experiencing a resurgence of the disease (14). In LMICs, there exists a significant deficit in the availability of accurate epidemiological data concerning pertussis. Research indicates that these regions should prioritize the implementation of polymerase chain reaction (PCR) testing for confirmation of pertussis due to its superior sensitivity, which is more likely to capture the actual burden of pertussis (14). Furthermore, there is a critical need for the establishment of a standardized monitoring system (31).

From 2020 to 2021, a notable decrease in the incidence of global pertussis cases was observed, primarily attributed to the impact of the COVID-19 pandemic. This decline can be elucidated through two primary pandemic-related factors. First, the widespread implementation of nonpharmaceutical interventions, including social distancing measures and the mandatory use of face masks (32, 33). Second, the disruption of routine vaccination sessions has had a profound impact on pertussis vaccination coverage (34). Notably, in April 2020, there was a global reduction of 33% in the administration of DTP vaccine doses (35). This significant decline in vaccination coverage may play a critical role in the current pertussis outbreak (35). However, after the pandemic, pertussis cases began to rebound in several countries (15). In the United Kingdom, the incidence of pertussis exhibited a dramatic increase, escalating from 0.12 cases per 100,000 individuals in 2022 to 38 cases per 100,000 individuals by 2024. Similarly, Australia experienced a significant rise in pertussis incidence, which surged from 1.83 cases per 100,000 individuals in 2022 to an alarming 214 cases per 100,000 individuals by 2024 (29). It has been suggested that the current situation may reflect a return to pre-pandemic pertussis patterns before the implementation of COVID-19 restrictions. Conversely, others argue that the sharp rise in cases supports the hypothesis of a new pertussis epidemic (36).

Accurately defining the global epidemiology of pertussis presents considerable challenges, particularly in contemporary contexts where its epidemiological landscape has evolved in complexity. Domenech de Cellès and colleagues examined the incidence trends of pertussis from 1980 to 2019 across 45 nations, utilizing data from the WHO. Their findings underscored significant heterogeneity in global pertussis incidence trends, revealing that this variability persists even in recent years within high-income countries that maintain robust vaccination coverage and adhere to multiple recommended booster schedules (37). The heterogeneity of the epidemiological dynamics of pertussis highlights its inherent complexity, which is shaped by various factors. One significant aspect is the variation in national reporting systems. Most countries where pertussis is recognized as a notifiable disease have established case-based national surveillance systems. However, the methods for monitoring and reporting have evolved. For example, in Japan, the National Center for Epidemiology of Infectious Diseases initiated pediatric sentinel surveillance for pertussis in 2017; and by 2018, it mandated laboratory confirmation for reports of the disease (38). Additionally, diagnostic methods and case definitions for pertussis vary across countries, including the clinical case definition of pertussis by the WHO, the requirement of laboratory confirmation, local case definitions, and the clinical diagnostic methods by physicians. These differences can affect the number of pertussis cases identified and reported (39). The variability in pertussis epidemiology is substantially driven by a range of factors. These include not only regional differences in historical and contemporary immunization strategies, but also variations in sociodemographic factors and patterns of social interactions. Moreover, the intricate interplay between the ecological conditions and the evolutionary developments of Bordetella pathogens further underpins the observed disparities (37). In summary, the worldwide epidemiological trends of pertussis result from a complex interplay of factors. These determinants include variations in disease surveillance systems, diagnostic methods, and immunization strategies, as well as differences in sociodemographic characteristics, patterns of social contact, and the impacts of the COVID-19 pandemic. A thorough understanding of these factors is essential for devising targeted, robust measures to effectively curb and prevent the global spread of pertussis.

Accurately delineating the global burden of pertussis presents a significant challenge within the field of public health. Factors such as under-reporting and under-diagnosis exacerbate this issue, particularly in adolescent and adult populations. In these age groups, pertussis often presents with atypical symptoms or is asymptomatic, leading to significant under-recognition and under-diagnosis (13). This phenomenon highlights the necessity for improved surveillance and diagnostic criteria to enhance the understanding and management of pertussis across all age groups. In the realm of pertussis diagnosis, laboratory culture and PCR are widely regarded as the gold standard methodologies (40). However, its atypical presentation in adolescents and adults has prompted a consensus among experts that serological assays may be more appropriate for evaluating the prevalence and burden of both recent and historical infections. Epidemiological studies support this view, with seroepidemiological surveys estimating incidence rates substantially higher than those reported through clinical surveillance (41). Nevertheless, serological diagnostics present certain limitations. First, there is considerable variability among commercial enzyme-linked immunosorbent assay kits due to differences in antigen composition and quality. Studies have shown that kits using mixed or unspecified antigens and lacking proper standardization yield inconsistent sensitivity and specificity (42). Moreover, the availability of reference antigens characterized by high purity remains a considerable challenge (43). Second, there is currently no globally recognized correlate of protection for pertussis, making interpretation of the results difficult (44). The European Union recommends the determination of specific antibodies against pertussis toxin (PT). However, there is no internationally accepted positive cut-off value. Reported diagnostic thresholds for anti-PT IgG vary, reflecting the absence of a uniform standard (45). These population-derived cut-off values necessitate continual reassessment in response to alterations in the vaccination schedule (45, 46). Finally, interpreting serology in recently vaccinated individuals is especially challenging. A high anti-PT IgG titer shortly after vaccination may not indicate infection at all, as current assays cannot distinguish vaccine-induced antibodies from those due to natural infection (45, 46). For instance, a household investigation linked to the DTP clinical trial in Sweden consistently observed seropositivity even when culture tests yielded negative results (47). Consequently, a comprehensive assessment of pertussis’ global burden requires a multifaceted approach, integrating clinical symptoms, laboratory diagnostics, and epidemiological data to achieve more accurate estimations.

### 2.2 Disease burden shifting from infants to older age groups

Historically, pertussis was considered as a vaccine-preventable childhood disease. Before the advent of the pertussis vaccine, it was estimated that up to 80% of the population contracted pertussis during childhood, with adult cases being rare (24, 48). Reports indicated that only 1–2% of all pertussis cases occurred in individuals aged 15 and above (49). However, recent epidemiological data indicate that a shift in the peak occurrence of the disease has transitioned from the infant population to older adolescents and adults (50). The evolving epidemiological landscape of pertussis is evidenced by case notification rates and seroprevalence data (51); however, the true burden of the disease among adolescents and adults remains uncertain due to under-recognition and under-diagnosis (52). The observed transformation in the incidence of pertussis among adolescents and adults may be attributed to waning immunity against *B. pertussis*, advancements in diagnostics and a relative increase in their proportion with a significant decrease in cases among infants following the vaccine’s introduction (52). Additionally, the transmission model has evolved; rather than infants primarily transmitting the disease to older individuals, adolescents and adults have increasingly emerged as the principal reservoirs and sources of transmission to infants (18, 39, 53). From 2000 to 2016, the proportion of pertussis among adolescents increased from 10% to 16% in the United States (30). Notably, 2004 was a peak year for pertussis outbreaks, during which adolescents represented 30% of all cases. Similarly, the 2010 outbreak highlighted significant disease prevalence among children aged 7–11 years (54). Data from the European Centre for Disease Prevention and Control indicated that in 2014, the highest incidence of pertussis occurred in <1-year-olds (51.6 cases per 100–000 population), followed by children aged 10–14 years (24.4 per 100000 population) and15-19-year-olds (19.7 per 100000 population) (55). More recently, during the 2023–2024 outbreak in Denmark, the incidence rate was the highest in the 3–4 months age group, recorded at 541.4 cases per 100,000 individuals, followed by the adolescent population, which had 405.4 cases per 100,000 individuals, with the highest increase (16). Furthermore, the latest pertussis outbreak in Spain primarily affected children aged 10–14 years, with 1,772.2 cases per 100,000 population (56).

A study conducted between 2006 and 2013 in the United States revealed that over 66% of pertussis infections in infants could be traced back to close family members. Notably, siblings accounted for 35.5% of the cases, followed by mothers at 20.6% and fathers at 10.0% (18). Additionally, surveillance data from Australia covering the period from 2008 to 2012 corroborated these findings, indicating that parents were responsible for 38.5% of pertussis infections in infants, while siblings constituted 35.4% (57). In fact, due to under-reporting and missed diagnoses, pertussis in adolescents and adults may be underestimated (39, 58). Therefore, pertussis prevention and control strategies should target all age groups, particularly adolescents and adults, to effectively reduce the risk of infection among infants.

Pertussis in adolescents and adults is generally not associated with severe illness or mortality. Nevertheless, numerous studies have shown that this population is the primary reservoir and source of infant pertussis infection. Infants are at the greatest risk of experiencing severe complications and mortality following infection (18, 39). Adolescents and adults often exhibit atypical clinical symptoms that may go undetected, yet they continue to serve as vectors for bacterial transmission. This concealed reservoir poses a significant threat to public health, as it has the potential to facilitate the onset of disease outbreaks (59, 60). Moreover, the mortality from pertussis-related hospitalizations in adults is relatively high (61), and complications such as apnea, insomnia, pneumonia, weight loss, urinary incontinence, fainting, and rib fractures may also occur (52). The susceptibility of older adults, particularly those with respiratory or chronic conditions, to pertussis is notably elevated, resulting in a higher incidence of hospitalization and mortality compared to younger individuals (52). Furthermore, the rise in reported pertussis cases among adolescents and adults can be attributed to multiple factors, prominently including the waning immunity that develops over time following the primary childhood vaccination (52). In light of this trend, the Global Pertussis Initiative recommended the expansion of pertussis vaccination strategies to incorporate a booster dose of the tetanus toxoid–reduced diphtheria toxoid–acellular pertussis (Tdap) vaccine for both adolescents and adults in 2006 (39). After the pertussis vaccine was introduced for adolescents in the United States in 2005, a marked reduction in pertussis incidence was observed in individuals between the ages of 11 and 18 years (62). Similarly, after the implementation of vaccination for high-school students in Australia in 2008–2009, a sustained reduction in adolescent pertussis cases was observed (63). Meanwhile, introducing Tdap for adults aged 25–39 years in France in 2008 significantly reduced pertussis incidence among adults. Studies conducted in the Paris area show that the incidence of pertussis in adults declined from 884 cases per 100,000 in 1999–2000 to 145 cases per 100,000 by 2008000ts (64). Notably, both periods coincided with national epidemic outbreaks (65).

## 3 Pathogenic mechanisms

Once *B. pertussis* is transmitted to humans through aerosolized particles, it colonizes the ciliated epithelial cells of the upper respiratory tract, causing inflammation, immune response activation, and host tissue damage (66). To efficiently colonize the respiratory tract and evade host immune defenses, *B. pertussis* secretes an array of virulence factors. These factors include various adhesins that facilitate attachment to host tissues and a repertoire of toxins that disrupt host cellular processes and immune function (66–68) (**Table 1**). Pertussis results from the coordinated interplay of these virulence factors. Adhesins such as filamentous hemagglutinin (FHA), pertactin (PRN), and fimbriae (FIM) play critical roles in facilitating the adhesion and sustained colonization of *B. pertussis* on epithelial cells, while toxins, such as PT and adenylate cyclase toxin (ACT), damage epithelial cells and modulate immune system activity, thereby promoting bacterial survival in the respiratory tract (68, 69). The expression of the genes responsible for these virulence factors is regulated by multiple systems, underlining the complexity of *B. pertussis* pathogenesis (68).

**Table 1:** Major virulence factors of *B. pertussis*.

Virulence factor [reference(s)]	Structure	Location	Component	Mechanism of action in pertussis
Toxins				
Pertussis toxin (PT) (85)	117 kDa; AB_5_-type exotoxin	Periplasm	Component of acellular vaccines alone or in combination	Ribosylates inhibitory G proteins and causes an increase in cyclic AMP
Adenylate cyclase toxin (ACT) (101, 222)	177 kDa; RTX toxin	Extracytoplasmic	Not a component of acellular vaccines	Converts intracellular ATP to cyclic cAMP and affects superoxide generation, immune effector cell chemotaxis, phagocytosis, and bacterial killing
Tracheal cytotoxin (102, 223)	9.2 kDa; Disaccharide-tetrapeptide monomer of peptidoglyca	Extracellular space	Not a component of acellular vaccines	Damages ciliated cells, inhibits DNA synthesis and extrusion of ciliated cells
Dermonecrotic toxin (104, 224)	160 kDa; Heat-labile toxin; typical A-B bacterial toxin	Cytoplasm	Not a component of acellular vaccines	Activates host GTP-binding protein Rho
Lipooligosaccharide (LOS) (225–227)	Endotoxin	Surface	Not a component of acellular vaccines	Causes a decrease in the number of neutrophils and causes coughing
Type III secretion system (T3SS) (228)	Needle-like structure	Cell envelope	Not a component of acellular vaccines	Secretes effectors and translocons
Adhesins				
Pertactin (PRN) (108, 109)	69 kDa; Autotransporter protein	Surface	Component of three- and five-component acellular vaccines	Binds to the ciliated tracheal epithelium and resists the clearance of neutrophils
Fimbriae (FIM) (113, 114, 229)	Fim2: 22 kDa; Filamentous protein	Surface projections	Component of acellular vaccines	Binds to tracheal epithelial cells, predominantly in trachea
	Fim3: 21.5 kDa; Filamentous protein			
Filamentous hemagglutinin (FHA) (115–117)	240 kDa; Filamentous protein	Cell wall	Component of most acellular vaccines	Binds to the ciliated tracheal epithelium and macrophage complement receptor 3 receptors, and promotes phagocytosis

### 3.1 Regulation of virulence factors in *B. pertussis*

The *Bordetella* virulence gene two-component system (BvgAS) in *B. pertussis* serves as the central regulator of virulence gene expression, modulating the activation of these genes in response to specific environmental signals (70, 71). BvgS is a multi-domain histidine sensor kinase, while BvgA is a response regulator protein. BvgS is activated at 37°C; using ATP, it auto-phosphorylates a conserved histidine residue in the HK domain, transferring the phosphate group to the receiver domain of BvgA (72). The activated phosphorylated BvgA then binds to the promoter region’s cis-acting sequences to activate the expression of virulence-activated genes (vags). Simultaneously, BvgR, encoded by vag, lowers the c-di-GMP levels by hydrolyzing it, which indirectly leads to the downregulation of virulence-repressed genes (vrgs) (71). This stage, known as the “Bvg^+^ phase” or “virulent phase,” is marked by the active expression of multiple virulence factors such as toxins and adhesins that are critical for *B. pertussis* to establish infection and exert pathogenic effects (73).

When *B. pertussis* is cultured in the presence of chemical modulators, such as magnesium sulfate or niacin, BvgS remains in a non-phosphorylated state. Under these conditions, the expression of vags is repressed, while the expression of vrgs is markedly enhanced (74). This shift in gene expression suggests that vrgs may have a substantial role in the aerosol transmission of *B. pertussis*, potentially influencing its pathogenicity and transmission dynamics in host populations (74, 75). At this point, the bacteria exist in the “Bvg^−^ or avirulent phase.” Studies suggest that the transition from the Bvg^+^ phase to the Bvg^−^ phase within host cells may promote survival and persistence within macrophages, with the surviving bacteria contributing to disease transmission (76). The identification of an “intermediate phase,” referred to as the “Bvgi phase,” is a notable development in understanding the pathogenicity of *B. pertussis* (77). During this phase, specific adhesins such as FHA are expressed, while the production of toxins is notably absent. This phase is likely instrumental in promoting the aerosol transmission of the pathogen (77). The BvgAS system facilitates the expression of virulence factors during the transitions between the Bvg^+^, Bvg^−^, and Bvgi phases, enabling *B. pertussis* to survive, persist, and spread in various ecological niches (76).

**Figure 2:**
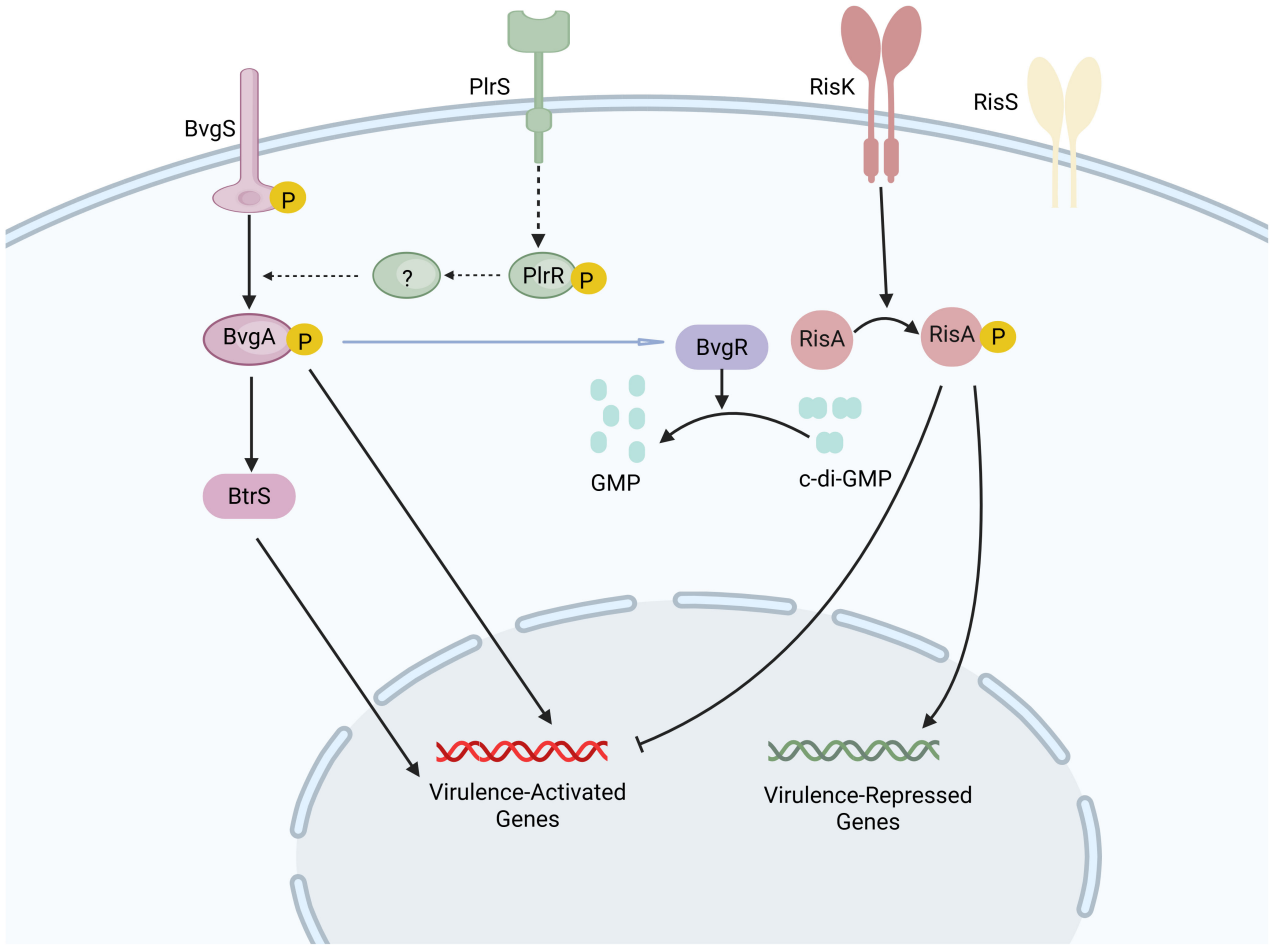
Regulatory systems of virulence factors in *B*. *pertussis*. At a temperature of 37°C, the sensor kinase BvgS is activated and transfers the phosphate group to its cognate response regulator BvgA through autophosphorylation. Upon phosphorylation, BvgA is activated, leading to the induction of virulence-activated genes (vags). The protein BvgR, encoded by vag, is capable of hydrolyzing cyclic di-GMP (c-di-GMP) into GMP. The response regulator RisA, which is phosphorylated by the sensor kinase RisK, binds to c-di-GMP in its phosphorylated form, triggering the expression of virulence-repressed genes while simultaneously repressing the expression of vags. The sensor kinase PlrS responds to carbon dioxide by phosphorylating the response regulator PlrR, potentially regulating genes required for bacterial colonization in the lower respiratory tract. The precise interaction between the PlrSR system and the BvgAS system remains to be fully elucidated. Created in Biorender.com.

It has been demonstrated that the virulence regulation of *B. pertussis* also involves several other systems (78). The regulator of intracellular signaling A and sensor kinase S two-component system (RisAS), an ortholog of the EnvZ-OmpR system, is involved in regulating responses to osmolarity, motility, and, in certain instances, virulence in various Gram-negative bacteria (79). A frameshift mutation in the RisAS alleles results in a truncated, nonfunctional RisS sensor kinase in *B. pertussis*. In this case, an alternative sensor kinase, RisK, is responsible for the phosphorylation of the response regulator RisA (80). High levels of c-di-GMP and phosphorylated RisA induce the expression of vrgs while suppressing the expression of vags (81). Additionally, the pyruvate kinase-like regulator and sensor two-component system (PlrRS) is believed to be essential for the persistence of *B. pertussis* in the lower respiratory tract. The PlrS sensor kinase is vital for the bacterial response to carbon dioxide, since it phosphorylates the response regulator PlrR (82). This phosphorylation event subsequently modulates the expression of genes essential for bacterial colonization within the lower respiratory tract. These genes may also encode proteins required for the sustained activity of the BvgAS system in the lower respiratory tract (82). In addition to the established two-component regulatory systems, sigma/anti-sigma factors may also modulate the expression of specific virulence factors. For instance, BvgAS activates the extracellular sigma factor BtrS and is responsible for the type III secretion system (T3SS) expression; while BtrA, acting as an anti-sigma factor, inhibits the activity of BtrS (83, 84). An overview of the regulatory systems of virulence factors in *B. pertussis* is depicted in **Figure 2**.

### 3.2 Major virulence factors of *B. pertussis*

PT is a unique and critical virulence factor of *B. pertussis* (85). It is a multi-subunit AB5-type protein toxin comprising one active subunit (A) and five binding subunits. It is secreted through a type IV secretion system encoded by the *ptl* locus. The A subunit exhibits ADP-ribosyltransferase activity, facilitating the covalent transfer of an ADP-ribose moiety from the cosubstrate NAD+ onto alpha subunits of inhibitory G proteins (85). This modification disrupts the inhibitory effect of inhibitory G proteins on adenylate cyclase, leading to increased cyclic adenosine monophosphate (cAMP) production (85). PT causes a range of systemic and local symptoms associated with pertussis. In infants and young children, the clinical presentation of pertussis is frequently marked by a significant increase in circulating white blood cells (86), particularly lymphocytes (87), a phenomenon typically indicative of a poor prognosis and closely related to the occurrence of pulmonary hypertension (88). Studies have shown that PT induces leukocytosis through various mechanisms (89), including downregulation of leukocyte adhesion molecules such as lymphocyte function-associated antigen-1 (90) and L-selectin (87, 91), as well as the suppression of chemokine receptor signaling that affects leukocyte migration (92). Furthermore, PT contributes to hyperinsulinemia and histamine sensitivity (93), and it facilitates the colonization of the respiratory tract by *B. pertussis* (94). During the initial phase of infection, PT targets alveolar macrophages, promoting *B. pertussis* infection. It also inhibits the production of cytokines and chemokines by alveolar macrophages, thereby reducing the recruitment and influx of neutrophils into the airways. Such inhibition undermines the early innate immune responses, thus enhancing bacterial colonization and persistence within the respiratory tract (95, 96). In the later stages of infection, PT exacerbates the inflammatory response in the lungs (97).

ACT, an immunogenic protein encoded by the *CyaA* gene, consists of an adenylate cyclase domain and a pore-forming repeats in toxin (RTX) domain (98). The adenylate cyclase domain becomes enzymatically active upon binding to calmodulin and subsequently catalyzes the conversion of ATP to cAMP within host cells, resulting in elevated intracellular cAMP levels (99). ACT interacts with neutrophils and macrophages through complement receptor 3, inhibiting their oxidative burst and phagocytosis, thereby facilitating *B. pertussis* infection (100, 101). Additionally, ACT induces macrophage apoptosis (99), enabling the bacteria to resist neutrophil-mediated clearance (67).

Tracheal cytotoxin works synergistically with lipooligosaccharide (LOS) to damage ciliated airway epithelial cells by inducing the production of pro-inflammatory mediators and nitric oxide (102). Furthermore, tracheal cytotoxin suppresses the chemotaxis of neutrophils, thus impairing optimal immune responses (103). Dermonecrotic toxin activates Rho GTPases in target cells, leading to skin necrosis in experimental animals (104).

LOS is a central glycolipid molecule on the outer membrane of *B. pertussis*. In *B. pertussis*, LOS triggers toll-like receptor 4 (TLR4) signaling, inducing the release of the cytokine interleukin 8 (IL-8) and tumor necrosis factor-alpha. This immunological activation is associated with a reduction in neutrophil counts localized at the site of infection within a few hours following infection in murine models (105). Moreover, LOS plays a critical role in bacterial colonization in the respiratory tract and nasal cavity (106).

T3SS apparatus is a sophisticated macromolecular injectisome that facilitates the direct translocation of effector proteins from the bacterial cytosol into the cytosolic compartment of host cells. T3SS is regulated by BvgAS. Although its role in pathogenesis remains unclear, its effector protein *Bordetella* type III effector A (BteA) has demonstrated potent cytotoxicity *in vitro* (107).

PRN is a 69-kDa autotransporter protein localized on the surface of *B. pertussis*; it has been demonstrated to mediate adhesion to epithelial ciliated cells (108) and to resist neutrophil-mediated clearance (109). In addition, PRN represents a primary target of the host antibody response and is a key component in the majority of aP vaccines. The widespread use of aP vaccines may have exerted a selective pressure on *B. pertussis*. Consequently, *B. pertussis* may have undergone evolutionary adaptations leading to the down-regulation of PRN expression or the generation of PRN variants that evade immune responses induced by vaccination (110). Studies suggest that the absence of PRN leads to increased immune activation and proinflammatory cytokine secretion (111). However, this increase in inflammation does not seem to result in more severe clinical manifestations (112). This phenomenon may be attributable to the bacteria’s ability to avoid immune responses induced by vaccines containing PRN, allowing the bacteria to circulate more efficiently in the population (112).

FIM of *B. pertussis* are members of the type I pili family. This bacterium produces two major fimbrial proteins, Fim2 and Fim3 (113), which play a critical role in facilitating initial bacterial interactions with airway epithelial cells. They also cooperate with FHA to suppress inflammation, thus promoting extended colonization of the respiratory tract (114).

FHA, encoded by *fhaB*, is a major adhesin molecule of *B. pertussis* (115). It is initially synthesized as a ∼370 kDa precursor protein (FhaB), which is processed into the mature ∼240 kDa FHA molecule by the serine protease subtilisin-like protease B1 (116). FHA is secreted through the two-partner secretion pathway (117) and plays a critical role in mediating bacterial adhesion to ciliated cells in the respiratory tract (118).

### 3.3 Importance of animal models in *B. pertussis* pathogenesis research

Developing suitable animal models has been challenging since *B. pertussis* is a strict human pathogen. Nevertheless, the availability of wild-type and transgenic strains has rendered them appealing as model organisms in disease research and vaccine development (75, 119). However, as *B. pertussis* is poorly infectious to murine hosts, the successful establishment of infection necessitates high numbers of *B. pertussis* directly to the lungs. In contrast, human infection typically begins with colonization and growth in the upper respiratory tract. Consequently, traditional mouse models may overlook the host’s mucosal defense mechanisms, which are crucial for understanding host-pathogen interactions related to transmission (120, 121). Recently, a newly developed mouse nasopharyngeal infection model has been shown to efficiently establish *B. pertussis* infection mimicking human disease, starting with colonizing the upper respiratory tract with low numbers of pathogens. The model may be helpful in the development of new and improved vaccines (121). Moreover, a recent baboon model has replicated various clinical manifestations of pertussis, including low-grade fever, paroxysmal cough, and lymphocytosis, while also illustrating that pertussis transmission can occur via contact and aerosolization pathways (122). Importantly, findings from studies employing non-human primate infection models have indicated that aP vaccination may prevent the development of disease symptoms. However, it does not prevent colonization or subsequent transmission (123). However, the use of non-human primate models presents ethical and cost challenges.

## 4 Prevailing explanations for pertussis resurgence

**Figure 3:**
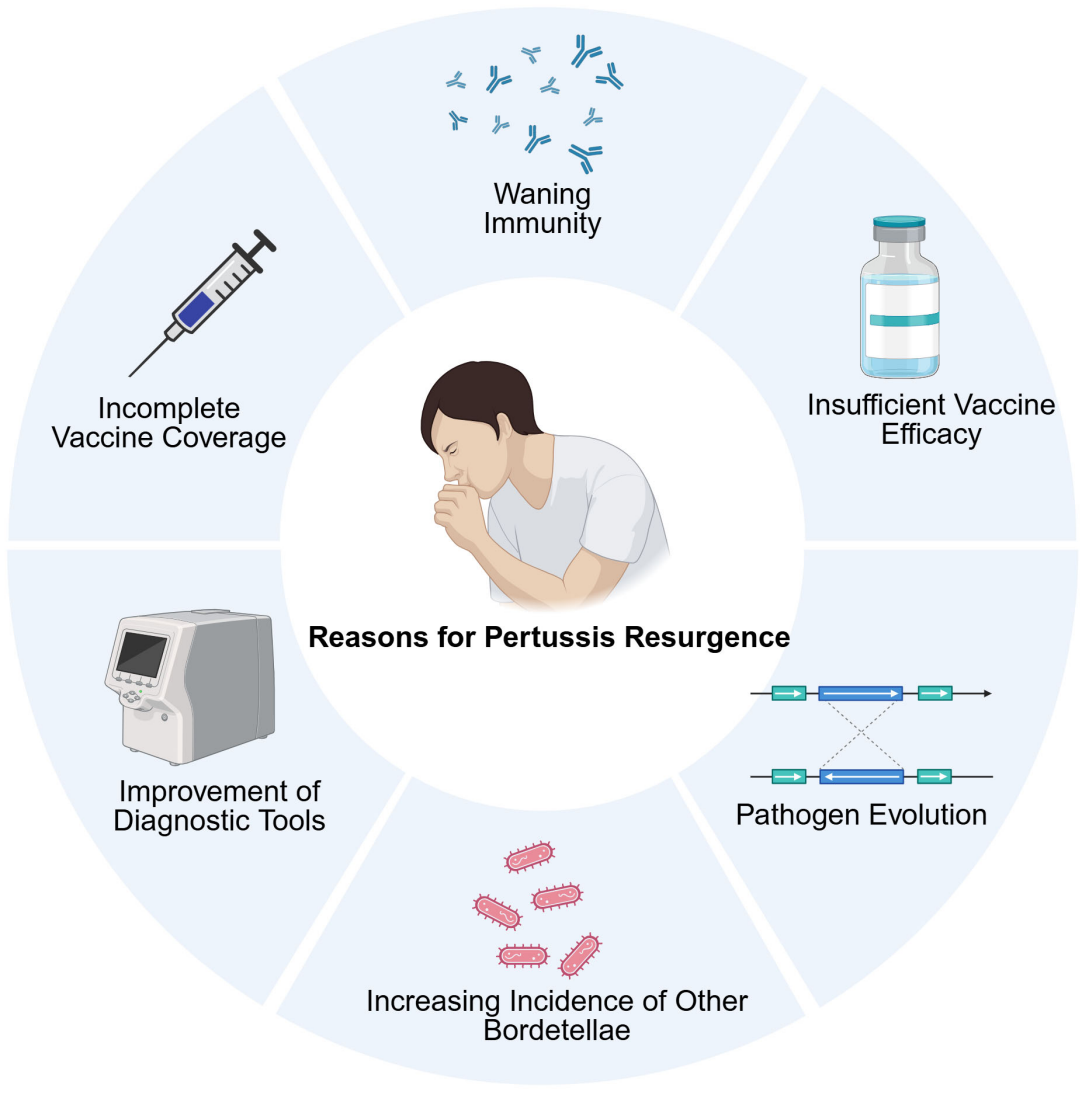
The main causes of pertussis resurgence, including waning immunity, the application of aP vaccines, the misdiagnoses of other *Bordetella* species, the improvement of diagnostic tools, and others. Created in Biorender.com.

Pertussis is endemic worldwide. Despite achieving a high level of vaccination coverage, numerous regions globally, including those utilizing wP vaccines, have experienced a resurgence of pertussis (37). This trend has prompted discussions regarding the underlying factors contributing to this phenomenon. The reasons for pertussis resurgence are controversial and multifactorial (**Figure 3**). This part attempts to explain the resurgence of pertussis by examining the prevailing evidence and associated phenomena. Waning of immunity is one of the hypothesized contributing factors to pertussis resurgence (124). Moreover, pathogen mutation has also been observed (125). Some studies have highlighted the potential role of genotype and phenotypic alterations in circulating *B. pertussis* strains (126). Other factors, such as the increased incidence of other *B.* species and improvements in diagnostic capabilities, may also contribute to the resurgence of pertussis to some degree (127–129).

### 4.1 Waning immunity and different immune responses

Immunity acquired through natural infection or pertussis vaccination is not lifelong, and immunity diminishes over time (130, 131). Studies demonstrate that immunity acquired from natural infection lasts longer than vaccination. However, the duration of protection following natural infection can vary among individuals, with more than 10% losing immunity within 10 years, while others may remain protected for over 30 years (132). The duration of protection provided by wP vaccines is similar to or slightly lower than that from natural infection (133). In contrast, the immunity provided by aP vaccines seems to exhibit a more limited duration of protection. A case-control study in Canada showed that the immunity of people who received the aP vaccine was significantly waning, with the vaccine effectiveness declining to 41% after more than 8 years after the last vaccination dose (134). A recent retrospective cohort study concluded no evidence of waning vaccine effectiveness for up to 4 years after five doses of the aP vaccine among 5–9 years old children (135). However, the study design can influence the estimation of the pertussis vaccine’s effectiveness, given variations in the control groups (136). Several studies have established data-based pertussis transmission dynamics models to investigate further the duration of protection conferred by aP vaccines. One such model estimated that the duration of protection could be as low as 5 years (137). In contrast, other modelling studies have suggested that aP vaccine-induced immunity may persist for several decades on average (138, 139). An explanation for the disparities in the estimated efficacy of aP vaccines lies in the intricacies associated with these vaccines and their interactions with concurrent immunizations. Research has indicated that the Bacillus Calmette-Guérin vaccination may enhance the protective efficacy against pertussis afforded by diphtheria, tetanus, and acellular pertussis vaccine (140).

**Figure 4:**
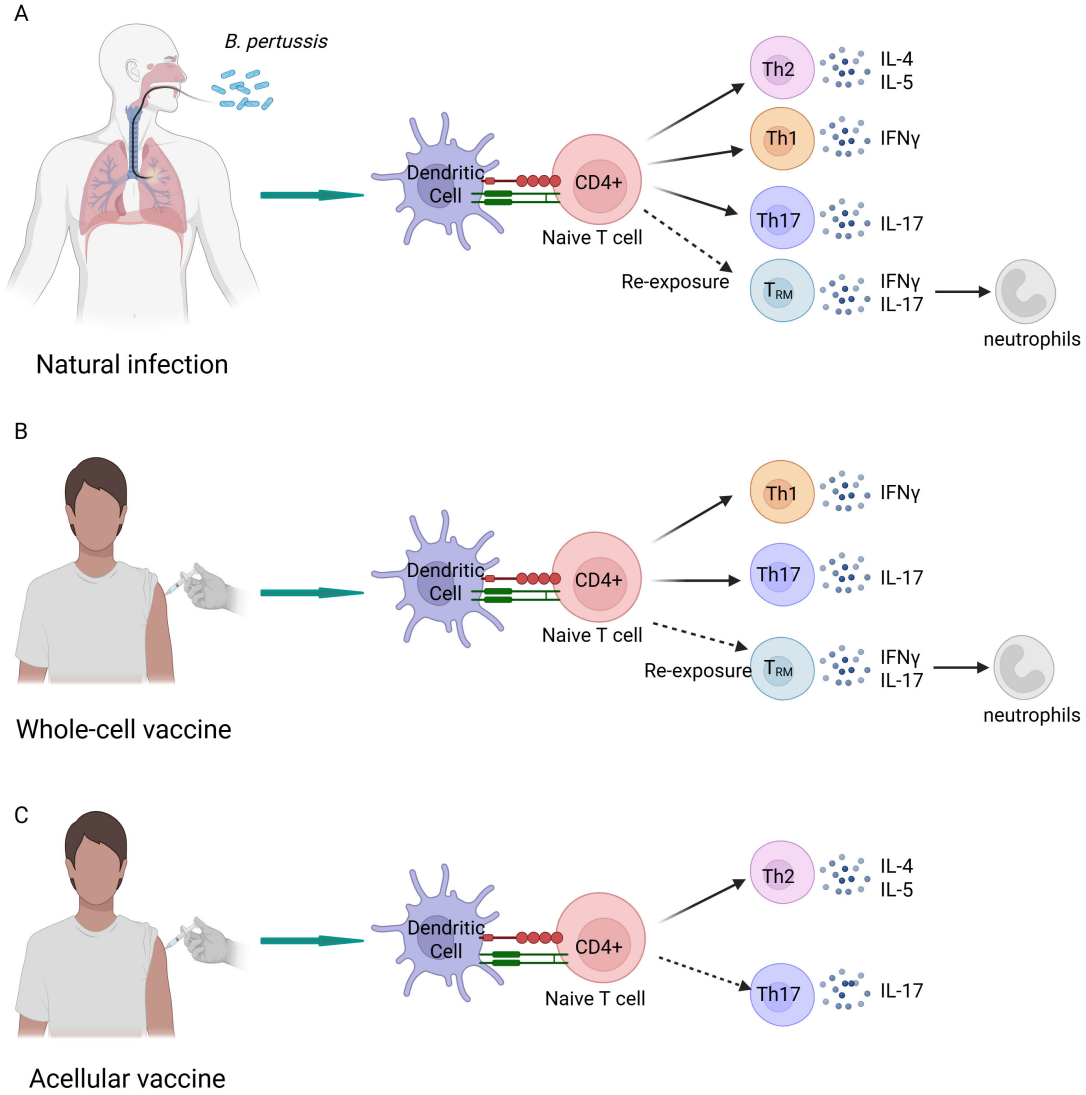
Differential immune responses to natural infection and immunization with whole-cell and acellular vaccines. **(A)** Natural infection elicits a cellular immune response dominated by T helper (Th) 1 and Th17 cells, while also inducing a Th2 cell-mediated immune response. Upon reinfection, CD4^+^ tissue-resident memory cells are activated, secreting interleukin (IL)-17A, which recruits neutrophils to the nasal mucosa. **(B)** The cellular immune response triggered by the whole-cell vaccines closely resembles that induced by natural infection; however, it does not predominantly involve Th2 cells. **(C)** The acellular vaccines predominantly induce a Th2 cell-mediated immune response, which is significantly different from the immune responses elicited by whole-cell vaccines and natural infection. Created in Biorender.com.

The immune response elicited by aP vaccines differs from that induced by natural infection and wP vaccination (141, 142) (**Figure 4**). Specifically, natural infection and wP vaccination induce the rapid proliferation of the CD4^+^ tissue-resident memory (TRM) T cells in respiratory tissues, which secrete interferon gamma and IL-17. This ability is crucial for the establishment of long-term immunity and effective bacterial clearance (143, 144). Regarding systemic immunity, natural infection and wP vaccination primarily induce T helper 1 (Th1) and Th17 cell-mediated immunity, leading to the secretion of interferon gamma and IL-17, respectively (123, 145). In contrast, aP vaccines are primarily linked to the induction of a Th2-biased immune response, which results in the elevated production of cytokines such as IL-4, IL-9, and transforming growth factor beta. This response is associated with the reduced production of IgG antibodies, which are crucial for neutralization and opsonization (123, 146). Animal models suggest that Th1 and Th17 cells play an important protective role in clearing *B. pertussis* and preventing reinfection, whereas the Th2 response has a shorter duration and limited protective effects. While it may prevent disease, it is less effective in reducing bacterial colonization (123, 147). This immune discrepancy persists even after multiple booster doses of aP vaccines (148, 149). The protective efficacy of acellular vaccines remains a topic of considerable debate. Recent studies employing non-human primate infection models have indicated that aP vaccination may prevent the development of disease symptoms but does not prevent colonization or subsequent transmission (123). It is important to interpret these findings with caution, as they are only derived from animal models of transmission. In contrast, much available epidemiological evidence indicates that aP vaccination may effectively block infection and transmission, forming herd immunity (150). The current epidemiological studies of pertussis present several limitations that warrant consideration. These include the incomplete detection of pertussis cases, the absence of a serological marker of immunity, and heterogeneity in diagnostic methods of surveillance (37). Additional rigorous epidemiological studies are needed to elucidate and clarify the dispute. Extending the human challenge model to individuals who have received aP vaccination will be crucial for enhancing our understanding of post-aP infections and reconciling discrepancies between findings from animal models and the epidemiological evidence in humans (37).

The debate surrounding aP vaccines underscores the complexity of pertussis resurgence (123, 150), which is likely attributable to a confluence of factors. Implementing aP vaccines appears to be an important factor in the resurgence of pertussis, but additional contributory elements also need to be considered and explored.

### 4.2 Pathogen evolution

*B. pertussis* has been evolving and is characterized by gene rearrangements and losses (151). Research indicates that such evolutionary dynamics have persisted throughout both the wP vaccination era and the aP vaccination era. Increasing evidence suggests that pathogen may adapt in response to vaccine-induced immunity, potentially undermining the effectiveness of aP vaccines (37). While the role of pathogen evolution in relation to the resurgence of pertussis remains to be fully elucidated, it remains worth exploring the phenomenon in the context of the pertussis resurgence (152).

Early serotyping systems primarily classified *B. pertussis* strains based on the presence of the heat-labile agglutinogen FIM (153, 154). Before widespread vaccination, many countries reported isolates of FIM2 and FIM2/3 (155, 156). However, after the implementation of wP vaccines, there was a significant shift in the serotype of isolates from FIM2 and FIM2/3 to FIM3. This change may have been due to the early vaccines lacking the FIM3 serotype (155). In the era of aP vaccines, studies have demonstrated that genes encoding antigens included in aP vaccines evolve at a markedly faster rate than genes encoding other nonvaccine surface antigens (157). Among these, the rise and widespread occurrence of PRN-deficient strains in countries employing vaccines containing PRN have been widely discussed (158, 159). Antibodies against PRN, induced by aP vaccination, mediate the elimination of *B. pertussis* (160, 161). Research indicates that the emergence of PRN-deficient strains results from selective pressure induced by aP vaccination (162). Subsequent studies indicate a correlation between the duration of administration of aP vaccines containing PRN and the observed frequency of PRN-deficient isolates within circulating populations (163). For instance, Japan exhibited an initial higher prevalence of PRN-deficient strains (164), although this has dramatically declined with the shift towards vaccines that do not include PRN (165). Similarly, surveillance studies indicate that in Denmark, where the vaccine used contains only PT, the isolation rate of PRN-deficient strains is very low (166). Besides the loss of the PRN gene, various PRN gene mutations have been identified, with the most common being the insertion of IS481, leading to PRN deficiency (167). It is hypothesized that the differential selection of PRN may stem from potential functional redundancy, longer-lasting antibody functionality against it, and its location near the surface membrane, which enhances effective complement fixation (9). Moreover, studies suggest that PRN-deficient strains may possess a selective advantage over PRN-expressing strains in populations vaccinated with aP vaccines (162).

PT is a critical factor in the pathogenesis of pertussis, and its expression is governed by its promoter (ptxP), which includes two main alleles: ptxP1 and ptxP3. The global prevalence of ptxP3 strains supplanting ptxP1 strains has emerged as a significant phenomenon observed across various countries (168, 169). Strains with the ptxP3 allele are more toxic than those with the ptxP1 allele because they promote more PT secretion, increasing the virulence of *B. pertussis* (125, 168). Nevertheless, a definitive conclusion remains unclear due to the limited sample size in the study and subsequent investigations have failed to offer confirmed evidence (37).

Moreover, macrolide resistance in *B. pertussis* has become a concern in recent years. Macrolides, such as erythromycin, are conventionally regarded as first line antibiotics for the treatment of pertussis, and early treatment can mitigate severe disease (170). Macrolide resistance has been increasingly reported in China (171). A pertinent study conducted in northern China reported that the proportion of highly virulent, macrolide-resistant strains increased dramatically from 42.9% during 2019–2021 to 100% in 2022–2023; the spread of these strains may be an important factor in the recent resurgence of pertussis epidemics in China (172). The primary resistance mechanism is a point mutation at position 2047 (A2047G) in domain V of the 23S rRNA gene of *B. pertussis* (171). In addition, the emergence of macrolide-resistant *B. pertussis* strains in Europe is equally concerning (173). In contrast, in the 2024 pertussis outbreak in Slovenia, no *B. pertussis* isolates were found resistant to macrolides (174). The emergence of macrolide-resistant *B. pertussis* strains presents a substantial challenge to the effective treatment of pertussis. While the widespread resistance in a particular region is a recent phenomenon, the long-term implications of this resistance on *B. pertussis* biology remain unclear. Surveillance of this issue is crucial during this period of uncertainty.

The evolutionary transformation of *B. pertussis* raises significant concerns; however, the precise implications for the recurrence of pertussis are still not fully understood. This uncertainty arises from the observation that, in certain countries where non-vaccine alleles have become prevalent, aP vaccines continue to demonstrate efficacy (152). Sustained genomic and epidemiological surveillance will be essential for tracking these evolutionary trajectories and refining evidence-based vaccination strategies.

### 4.3 Other contributing factors

In addition to *B. pertussis*, other *Bordetella* species, including *Bordetella parapertussis* and *Bordetella holmesii*, also have been implicated in pertussis-like syndromes in humans (66). Recently, these non-*B. pertussis* species have been detected in humans with increasing frequency. Some experts have posited that the misidentification of non-*B. pertussis* species as pertussis may have played a significant role in the epidemics (127, 175). During a pertussis outbreak in Ohio in 2010, 32% of patients diagnosed with *B. pertussis* infection were found to have *B. holmesii* infection (176). This misdiagnosis occurs because the IS481 sequence, which is commonly used in standard PCR for diagnosis, is present in the genomes of both *B. pertussis* and *B. holmesii* (177); consequently, the prevalence of these non-*B. pertussis* species increases, and misdiagnosis of pertussis due to diagnostic limitations and insufficient laboratory expertise in identifying other *Bordetella* species may contribute to the resurgence of pertussis. Recent advancements in diagnostic methodologies have led to multiplex quantitative polymerase chain reaction kits for the detection of *Bordetella*. These kits enable the identification of IS481 for *B. pertussis*, pIS1001 for *B. parapertussis*, and hIS1001 for *B. holmesii* (178). Such innovations are poised to enhance the ability to quantify and analyze trends in the incidence of *B. pertussis, B. parapertussis*, and *B. holmesii* (178). Furthermore, improvements in disease awareness and diagnostic tools (128), as well as insufficient vaccine coverage in certain regions (129), may be potential factors contributing to pertussis outbreaks.

## 5 Strategies to address pertussis resurgence

**Figure 5:**
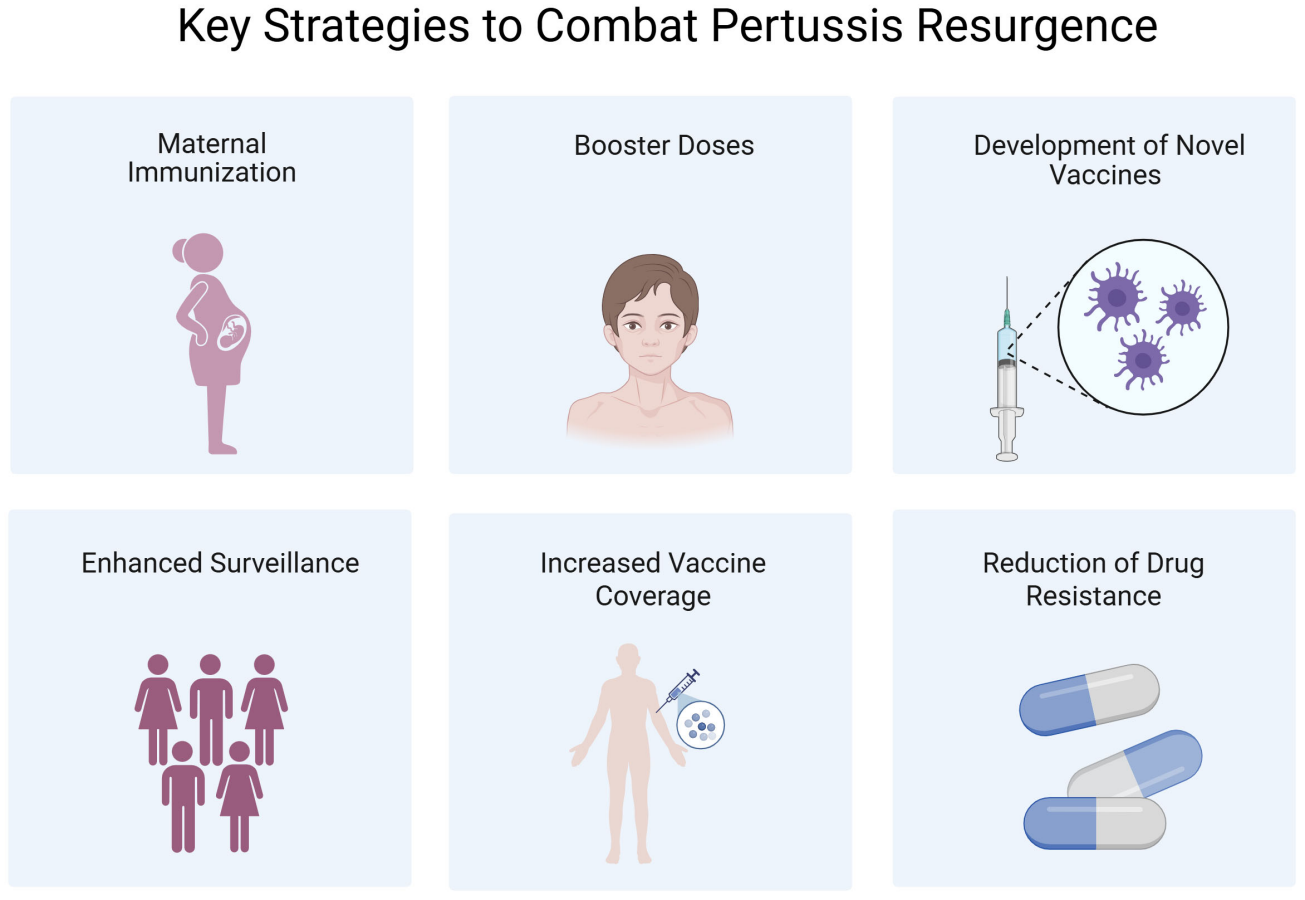
The key strategies to combat pertussis resurgence. Created in Biorender.com.

A growing body of evidence supports a multifaceted response to the global resurgence of pertussis. Vaccination remains the most cost-effective preventive measure. Although vaccine-induced immunity wanes over time, timely immunization substantially reduces the incidence of severe disease and pertussis-related mortality, especially among infants. Consequently, every country—particularly LMICs—should prioritize achieving and sustaining high vaccination coverage. Beyond maintaining coverage, effective countermeasures include optimizing vaccination strategies, developing next-generation vaccines, and strengthening genomic and epidemiological surveillance. A summary of these strategies is depicted in **Figure 5**.

### 5.1 Optimization of vaccination strategies

Newborns whose systems are not yet fully developed and who have not reached the age for vaccination are at a high risk for pertussis, especially showing the most severe symptoms and even mortality (28). To address this public health issue, many countries have implemented the Tdap vaccination strategy for pregnant women (179). This strategy causes the mother to produce high levels of antibodies, which are transferred to the fetus via the placenta. It provides passive protection during the newborn’s early life before they receive their vaccinations (180). This approach has been shown to significantly decrease the incidence of pertussis among infants in their first two months of life (181, 182). However, the optimal timing for maternal Tdap vaccination remains a subject of debate. Data collected in the United States suggest that vaccination in the late stages of pregnancy may be more effective than vaccination in the early or mid-pregnancy stages (183). However, another study indicates that vaccination in the second trimester significantly increases neonatal antibody levels, possibly due to a longer interval between vaccination and delivery (184). In the United Kingdom, it is recommended to administer the vaccine between 16 and 32 weeks of pregnancy, while the vaccination window in the United States is between 27 and 36 weeks of gestation (185). Although the timing of maternal vaccination varies internationally, all evaluated strategies have demonstrated significant effectiveness in reducing *B. pertussis* infections in infants. Epidemiological estimates suggest that laboratory-confirmed pertussis cases in unvaccinated neonates have decreased by 70–95% in both the United Kingdom and the United States (186, 187). Notably, some studies suggest that maternal antibodies may influence the infant’s initial immune response. Indeed, infants born to vaccinated mothers mount a weaker immune response to the primary pertussis vaccine than those born to unvaccinated mothers (188). However, epidemiological data indicate that the risk of pertussis after the initial vaccination does not increase for infants born to vaccinated mothers (186, 189). Moreover, a recent modeling study showed that maternal vaccination is highly beneficial in the long term (190). Based on the current evidence, maternal immunity appears to be cost-effective preventive strategy, offering effective protection during the critical window before infants can be vaccinated.

As previously mentioned, the protective effect of aP vaccines wanes over time, making adolescents and adults potential sources of pertussis transmission, particularly to infants who have not completed their full vaccination schedule (191). Therefore, several countries have incorporated adolescent pertussis booster immunizations into their national immunization programs. In the United States, introducing Tdap vaccination for adolescents in 2005 resulted in a substantial reduction in pertussis cases among those aged 11–18 years old (62). In addition to adolescent immunization efforts, certain countries advocate for routine booster vaccinations in the adult population at intervals of approximately ten years (192). Existing epidemiological evidence indicates that the protective effect of Tdap boosters is initially high but gradually wanes in adolescents and adults (193, 194). Moreover, the immune response to booster depends on the host’s vaccination history, especially the type of vaccine used for primary immunization. Individuals who received the wP vaccine for primary immunization generate stronger antibody and memory B cell responses following booster administration (195). Booster vaccination in these age groups helps reduce their disease burden and the risk of direct transmission to susceptible individuals, particularly unvaccinated infants.

### 5.2 Development of novel vaccines

As discussed above, given the debate over the duration and effectiveness of aP vaccine-derived protection, developing novel, highly effective pertussis vaccines has become a critical research focus for controlling this disease. Research teams are advancing vaccine improvement strategies from multiple perspectives, such as the development of low-reactogenic wP vaccines, optimization of the immunogenicity of existing aP vaccines, and innovative exploration of novel vaccine formats including outer membrane vesicle (OMV) vaccines and live-attenuated pertussis vaccines (**Table 2**).

**Table 2:** Comparison of the next-generation pertussis vaccines.

Vaccine type [reference(s)]	Key features	Immune response	Current status	Main challenges
Low-reactogenic wP vaccines (196, 197)	Broad antigen profile; modified to reduce LOS toxicity	Th1/Th17	Animal studies and early-phase clinical trials	Balance between reduced reactogenicity and maintained efficacy
Improved aP vaccines (201, 207)	Includes novel antigens/adjuvants for broader immune response	Th1/Th17	Animal studies and completed Phase II/III trial	Higher production cost
Pertussis OMV-based vaccines (211, 212)	Naturally contains multiple antigens and adjuvant properties	Th1/Th17/Th2	Animal studies	Endotoxin level and different antigen patterns resulting from OMV isolation techniques
Live-attenuated pertussis vaccines (BPZE1) (219)	Intranasal live vaccine; mimics natural infection	Th1/Th17/mucosal IgA	Completed phase IIb trial	Applicability to infants and young children; public acceptance

LOS, a key component of wP vaccines, exhibits endotoxin activity and is considered one of the major factors contributing to adverse reactions associated with wP vaccination (196). Some research aims to reduce or modify LOS to mitigate endotoxin activity and, consequently, reduce reactogenicity (197).

While aP vaccines exhibit good safety profiles, they contain a limited number of antigens, typically 1–5 purified antigens, which results in a narrow pertussis-specific immune response (198). This limitation may also contribute to immune pressure (162). Therefore, incorporating additional antigen components could effectively enhance the current immune protection and reduce the impact of immune selection pressure. For example, supplementation of aP vaccines with ACT at optimal doses has been demonstrated to elicit anti-ACT antibody production, facilitate a shift from a Th2-dominated response to a mixed Th1/Th2 response, and enhance protective immunity (199). PT, another major virulence factor of *B. pertussis*, is included in all current aP vaccines in its chemically detoxified form. However, chemical detoxification reduces the ability of the full toxin to bind to target cells and weakens key activities, including antigen presentation and cytokine secretion by antigen-presenting cells, thereby diminishing its adjuvanticity (200). Conversely, genetically detoxified PT is safe and retains the complete quaternary structure and cell-binding ability of the full toxin, preserving its adjuvanticity (200). It has been shown that genetically detoxified PT induces better and more durable immune responses compared to vaccines containing chemically detoxified PT (201).

Currently, the efficacy of aP vaccines with aluminum as the adjuvant is insufficient, as their mechanism primarily induces a Th2/Th17-biased immune response. However, effective clearance of *B. pertussis* requires the establishment of a robust Th1/Th17 immune response and the formation of long-lasting immune memory. This difference in mechanisms may lead to a limited protection duration with the current aP vaccines (202). Therefore, novel adjuvant systems that can effectively stimulate a potent Th1/Th17 immune response are needed.

TLRs are transmembrane pattern recognition receptors predominantly expressed on innate immune cells. TLR agonists can link innate immune responses with adaptive immunity, making them helpful in enhancing and accelerating early immunity induced by aP vaccines (203). Cytosine–guanine (CpG) motif-containing oligodeoxynucleotides can activate TLR9 signaling, leading to a robust Th1 response in both murine and human systems (204, 205). Importantly, experimental aP vaccines formulated with CpG have been shown to induce Th1 and Th17 responses as well as IgG2 antibody responses in mice, offering significant protection against pulmonary infection (202). Likewise, the presence of SMIP-7.10, a synthetic TLR7 agonist, in aP vaccines with alum as the adjuvant has been demonstrated to enhance the Th1 and Th17 immune responses specific to *B. pertussis*, thus improving the vaccine’s efficacy against an aerosolized pertussis attack (206). Compared to alum-adjuvanted aP vaccines, an aP vaccine formulated with LP1569, a TLR2 agonist, has revealed enhanced protection against lung and tracheal infections in mice, along with a strong Th1 and Th17 response specific to FHA (207). The investigation revealed that c-di-GMP, used as a mucosal adjuvant in ap vaccines, elicited enhanced antigen-specific antibody production and pronounced robust Th1 and Th17 immune responses. Additionally, this adjuvant facilitated more efficient bacterial clearance within the respiratory tract (208).

Outer membrane vesicles (OMVs) are spherical structures derived from Gram-negative bacteria’s cell membranes; they comprise outer membrane proteins, nucleic acids, and other substances (209). Due to their strong immunogenicity, OMVs have been developed in pertussis vaccines. Pertussis OMV vaccines present multiple bacterial antigens from natural structures, inducing comprehensive immune responses while avoiding the risk of excessive inflammation that is present with wP vaccines (209). In murine models, parenteral administration of pertussis OMV vaccines elicits Th1/Th17/Th2 responses in CD4^+^ TRM cells, providing stronger protection than aP vaccines and comparable protection to wP vaccines, while demonstrating an improved safety profile (210–212). Furthermore, the coadministration of pertussis OMVs with diphtheria-tetanus toxoids in murine models elicits long-lasting immune protection for up to 9 months and provides better resistance to PRN-deficient strains (213).

Locht et al. genetically engineered a live attenuated *B. pertussis* strain, BPZE1, which has the dermonecrotic toxin gene deleted, the amount of tracheal cytotoxin reduced to background levels, and complete inactivation of PT. In mouse models, BPZE1 effectively colonized the respiratory tract with low pathogenicity; and after a single intranasal dose, it induced better protection than aP vaccines (198). Additionally, mouse studies have shown that BPZE1 induces both antibody production and Th1/Th17 responses, generating long-term protective immunity against *B.* pertussis (214, 215). Moreover, local secretory immunoglobulin A and IL-17 induced by BPZE1 have been shown to protect the nasal and lung tissues of murine models from infection (216). BPZE1 is currently undergoing clinical development and has completed two Phase I trials. The findings indicate that the vaccine is safe and immunogenic in adults (217, 218). A subsequent phase IIb trial suggests that the vaccine induces robust and sustained pertussis-specific mucosal immunoglobulin A responses (219). BPZE1 has been evaluated for safety and immunogenicity in school-age children between 6 and 17 years of age in a phase IIb trial [ClinicalTrials.gov Identifier: NCT05116241]. Furthermore, BPZE1 has exhibited a favorable safety profile across multiple preclinical and clinical studies. Preclinical studies utilizing murine models have demonstrated that BPZE1 does not induce weight reduction, lung pathology or mortality (220). In a juvenile baboon model, BPZE1 was also found to be safe (221). Clinical trials conducted to date have not revealed any concerning safety signals associated with BPZE1 (217, 219). However, the forthcoming Phase III trials, alongside subsequent post-marketing surveillance, will be critical for validating both the safety and immunogenicity of BPZE1 in broader populations.

## 6 Conclusion

Despite comprehensive infant vaccination coverage in numerous countries, pertussis continues to pose a substantial public health challenge globally. Since the 1980s, even amid increasing vaccination rates, the incidence of pertussis has been on the rise, with a notable shift in the peak of incidence from infants to adolescents and adults, who have emerged as primary reservoirs of infection for the vulnerable infant population. Under-recognition, inadequate diagnosis, and systemic under-reporting have led to a significant underestimation of the true incidence of pertussis.

Global findings reveal considerable variability in disease trends across different nations, underscoring the complexity of the epidemiology of pertussis. The resurgence of pertussis may be associated with the widespread use of aP vaccines, pathogen evolution, and the increased incidence of other *B.* species. In response to this concerning trend, several countries have enacted interventions such as vaccination of pregnant women and embarking on the development of new vaccines. These initiatives have yielded some positive outcomes.

However, changing vaccination strategies is only a temporary measure, and it is essential to improve the efficacy of current pertussis vaccines and develop new vaccines to prevent and control *B. pertussis* infections. Inducing durable Th1- and Th17-polarized CD4+ TRM cells is critical in next-generation vaccine development. The recently developed vaccines have demonstrated the potential to enhance cellular immunity in preclinical models or early-phase clinical trials. Nevertheless, the path to market approval remains challenging. Vaccine development requires time, significant financial investment, and human resources. Furthermore, current protective efficacy assessments are mostly confined to preclinical models, and comprehensive safety profiles have yet to be fully characterized. Consequently, establishing these vaccines’ efficacy and safety profiles through rigorous clinical trials presents a significant challenge that must be overcome to achieve successful market authorization. Currently, pertussis vaccines are combined with tetanus and diphtheria vaccines, and no standalone pertussis vaccine is being utilized widely. Therefore, it is crucial to ascertain that this change does not affect immunity to tetanus and diphtheria in the short and long term. However, a potentially more effective long-term goal should be to replace the aP vaccines in the existing pediatric vaccine combinations with an easily administered nasal pertussis vaccine. Such a vaccine could facilitate the promotion of TRM cells, thereby providing sustained protective immunity at both the nasal mucosa and pulmonary levels.

In addition, several unresolved scientific questions and academic controversies regarding pertussis require further investigation. The absence of appropriate animal models has resulted in a gap in the comprehensive understanding of the pathogenesis of pertussis. Recent advancements, including the murine nasopharyngeal infection model and the baboon infection model, provide crucial insights into infectious disease mechanisms; however, there are ethical and cost challenges. Moreover, the emergence of PRN-deficient strains in multiple countries has sparked considerable debate over their potential impact on disease severity. While current evidence does not definitively associate PRN-deficient strains with more severe clinical outcomes, their increasing prevalence and potential fitness advantages in the immunity induced by aP vaccination require a more in-depth investigation. Another unresolved issue concerns the optimal timing of maternal immunization. Although various recommendations exist, further high-quality evidence is needed to determine the most effective pregnancy vaccination period.
